# Protein engineering approaches for antibody fragments: directed evolution and rational design approaches

**DOI:** 10.3906/biy-1809-28

**Published:** 2019-02-07

**Authors:** Merve ARSLAN, Dilara KARADAĞ, Sibel KALYONCU

**Affiliations:** 1 İzmir Biomedicine and Genome Center , İzmir , Turkey; 2 İzmir Biomedicine and Genome Institute, Dokuz Eylül University , İzmir , Turkey

**Keywords:** Antibody, antibody fragment, directed evolution, rational design, protein engineering, phage display, yeast surface display, afinity, biophysical properties

## Abstract

The number of therapeutic antibodies in preclinical, clinical, or approved phases has been increasing exponentially, mostly due to their known successes. Development of antibody engineering methods has substantially hastened the development of therapeutic antibodies. A variety of protein engineering techniques can be applied to antibodies to improve their afinity and/or biophysical properties such as solubility and stability. Antibody fragments (where all or some parts of constant regions are eliminated while the essential antigen binding region is preserved) are more suitable for protein engineering techniques because there are many in vitro screening technologies available for antibody fragments but not full-length antibodies. Improvement of biophysical characteristics is important in the early development phase because most antibodies fail at the later stage of development and this leads to loss of resources and time. Here, we review directed evolution and rational design methods to improve antibody properties. Recent developments in rational design approaches and antibody display technologies, and especially phage display, which was recently awarded the 2018 Nobel Prize, are discussed to be used in antibody research and development.

## 1. Introduction

Hundreds of therapeutic antibodies and their derivatives are being manufactured and tested in clinical trials. Currently, there are more than 65 monoclonal antibodies approved on the market for the treatment of various diseases, mostly cancer. The rate of antibody therapeutics receiving their first approvals has been increasing over the last decade. Last year, 10 antibodies were approved in either the European Union or the United States and this number is expected to increase in the upcoming years (Kaplon and Reichert, 2018) .

The first technology that was used to produce therapeutic antibodies was mouse hybridoma technology (Frenzel et al., 2017) . With this technology, therapeutic monoclonal antibodies (mAbs) are obtained via the fusion of murine B cells and myeloma cells. However, there are some limitations in the use of these mAbs in humans, especially the immune response against murine mAbs (human antimouse antibody response) (Qin and Li, 2014) . To overcome this problem, several approaches were developed by utilizing recombinant DNA technology, such as chimerization (replacement of the constant regions of the murine antibodies with homologous human sequences), which generally reduces the afinity and deteriorates biophysical properties of mAbs. eThrefore, it is essential to apply afinity maturation and protein engineering approaches after this process. More importantly, there are known reproducibility problems related to the hybridoma technique where sequence information is lost and features of mAbs cannot be improved with many available in vitro systems (Bradbury and Pluckthun, 2015) .

Approximately 90% of approved antibody drugs are full-length (IgG) and the rest are antibody fragments (mostly Fab formats), where all or some parts of constant regions are eliminated while the essential antigen binding region is preserved. It is very well known that antibody fragments usually show similar binding properties as their full-length versions with even better biophysical properties (Nelson, 2010) . Compared to full-length antibodies, antibody fragments have many advantages for therapeutic This work is licensed under a Creative Commons Attribution 4.0 International License. use: (i) lower immunogenicity due to lack of constant regions, (ii) higher tumor penetration, (iii) cheaper and larger scale production with bacteria, and (iv) availability of various in vitro display technologies to improve several characteristics of antibodies. Today, the number of antibody fragments in clinical trials and on the market is increasing faster than before due to their advantages. Because most of the directed evolution approaches are only available for antibody fragments, improvement of fulllength antibodies is usually conducted in their antibody fragment format, and then those improved fragments are converted back to full-length antibody format (Xiao et al., 2017) .

Protein engineering techniques such as directed evolution and rational design approaches to discover and/or improve antibodies are becoming more popular both in the biopharmaceutical industry and research environments. Applying these techniques in the early discovery phase is important because it is high-throughput and there is full control of protein sequence during the development phase of biotherapeutics.

## 2. Antibody display technologies as directed evolution approaches

For the past 40 years, hybridoma technology has been used extensively to produce traditional monoclonal antibodies for research and diagnostics. Recently, a number of advanced methods called display technologies have emerged as fast and high-throughput alternatives. Phage display technology is the first radical in vitro approach that allowed to produce human antibodies without any need for immunization. In this technique, antibody fragments are fused to a capsid protein of the phage and thus expressed on the surface of the virus (García Merino, 2011; Chiu and Gilliland, 2016) . Although phage display is the most common antibody display technique, today several recombinant display technologies are available and basically classified in two categories: in vitro display technologies (phage display, ribosome-mRNA display) and in vivo display technologies (bacterial, yeast, and mammalian cell-surface display) (Sergeeva et al., 2006; Harel Inbar and Benhar, 2012; Brodel et al., 2018) .

## 2.1. In vitro display technologies

### 2.1.1. Phage Display

The phage display technique was first discovered in 1985 by George P Smith, who was one of three recipients of the 2018 Nobel Prize in chemistry for this discovery (Smith, 1985) . This was an important step to develop new approaches for generation of mAbs. In this technique, a protein gene is fused to a gene encoding a capsid protein of the virus and the fused gene is inserted into a single-stranded DNA of the phage (Karimi et al., 2016; Ledsgaard et al., 2018) . Basically, two types of capsid proteins are preferred; the first one is pIII that allows to fuse larger proteins and the other one is pVIII. The most commonly used phages for phage display are the filamentous ones (M13, Fd, and f1), which are in the Ff family and have the ability to infect only the strains of Escherichia coli containing F conjugative plasmids (Li and Caberoy, 2010; Loset and Sandlie, 2012; Karimi et al., 2016; Gustafson et al., 2018; Kiguchi et al., 2018; Ledsgaard et al., 2018; Teixeira and GonzalezPajuelo, 2018) .

Two different application systems have been developed for phage display. In the first system, a protein sequence is used as an insert and is fused to a capsid gene of the virus. With this system, the desired protein is expressed within the genome of the virus. In the other more preferred system, a different plasmid called a phagemid is used and the expression of the desired protein is separated from the phage replication. Phagemids also include replication origins of E. coli and a phage, a specific selection marker, and specific tags that help detection and purification of the desired protein (Li and Caberoy, 2010; Loset and Sandlie, 2012; Teixeira and Gonzalez-Pajuelo, 2018) .

The phage display technique was first applied for the variable fragments of antibodies and many different antibody fragment formats have been displayed by this technique. The antibody fragments that are displayed by this technique are usually scFv (a single-chain variable fragment) or Fab (antigen-binding fragment), and nowadays the most popular ones are VH (nanobody, heavy variable domain of the antibody) (Teixeira and GonzalezPajuelo, 2018) . It is easy to convert these fragments to full-length antibodies by recombinant DNA technology, if needed.

**Figure 1:**
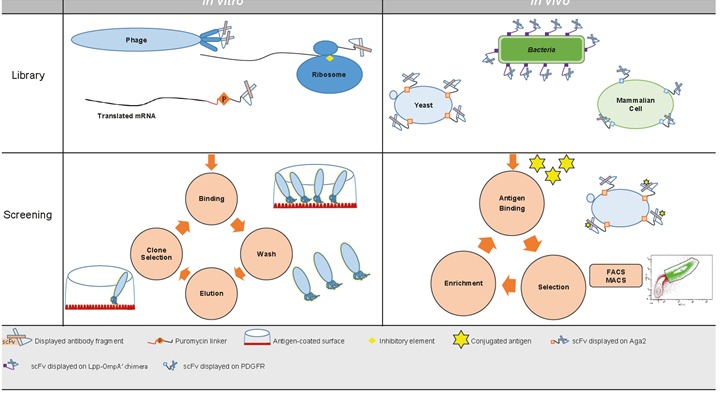
Antibody display technologies. General schematic for in vitro and in vivo display techniques.

The phage display technique is carried out by a process of in vitro repeated cycles typically named biopanning or phage display selections (Figure 1). This process includes the following steps: (1) incubation: binding of the antibody library repertoire to the antigen; (2) washing: elimination of the nonspecific binders; and (3) elution and amplification: obtaining antibodies binding to the antigen specifically for further cycles or for screening. Although in the first cycle of biopanning the whole antibody repertoire is exposed to the antigen, depending on the fragment type of the antibody and the phage display, 2–4 cycles of selections are generally performed to enrich the specific binders. Evaluation of the success of each cycle of the process and enrichment is possible by comparing the phage titers after elution steps against a blank that does not include antigen, or alternatively it can be tested by ELISA (enzyme-linked immunosorbent assay) (Hairul Bahara et al., 2013; Chan et al., 2014; Ledsgaard et al., 2018; Teixeira and GonzalezPajuelo, 2018) .

The most powerful advantages of phage display are its small size and high diversity (antibody libraries up to 1011 clones), which allow to obtain antibody fragments with the desired afinity and biophysical properties. Also, this technique is preferred in both research areas and the biopharmaceutical industry due to its library diversity, ease of use, and low cost (Liu et al., 2017; Teixeira and Gonzalez-Pajuelo, 2018) . For example, belimumab (market name Benlysta) was discovered and improved by phage display and it is used to treat adults with active systemic lupus erythematosus (Stohl and Hilbert, 2012) . As a better known example, adalimumab (market name Humira) was discovered by phage display and it is widely used for rheumatoid arthritis treatment (Bain and Brazil, 2003). The number of antibodies discovered and/or optimized by phage display has been exponentially increasing over the last decade due to the many advantages listed above (Nixon et al., 2014) .

### 2.1.2. Ribosome and mRNA display

Ribosome and mRNA display techniques are cell-free and this feature separates them from other display platforms. They have high molecular diversity (antibody libraries ranging from 1012 to 1014 clones) and enable isolation of antibodies that show afinities at pM level. Both techniques include the same basic features, such as in vitro transcription and translation steps (Harel Inbar and Benhar, 2012) .

Ribosome-display technology was first reported in a patent application in 1991. While the mRNA encoding antibody library is translated in vitro, the translated scFv peptide and corresponding mRNA remain attached to the ribosome (Figure 1). By this means, the peptide– ribosome–mRNA (PRM) complex can be selected along with the sequence information of the desired antibody by afinity purification techniques. The most powerful aspect of this technique is its large size of library, which is not limited by the cell transformation efficiency. On the other hand, the ribosome amount and the existence of unrelated mRNA molecules are the main limitations of this technique (Hanes and Pluckthun, 1997) . Groves et al. compared phage display and ribosome display to generate scFvs to a specific antigen (Groves et al., 2014) . They found that scFvs afinity-matured by ribosome display had more structural diversity in the HCDR3 and VH-VL interface regions.

In the mRNA-display technique, first the antibody DNA library is transcribed to mRNA. Then mRNA is ligated to a linker, which is a DNA sequence linked to puromycin. eThreaeftr, the mRNA-linker-puromycin complex is translated. Puromycin first binds to the A-site of the ribosome, then attacks the P-site and the nascent peptide is transferred to puromycin, resulting in the mRNA-linker-puromycin-antibody fragment complex. The complex is then reverse-transcribed and the selection process is performed. After the selection step, ss-DNA is obtained by hydrolyzing the complementary mRNA via high pH, and the desired DNA sequence is amplified by PCR (Figure 1) (Sergeeva et al., 2006; Jijakli et al., 2016; Liu et al., 2017) . One of the limitations of this technique is low efficiency of mRNA-protein conjugates. Nagumo et al. overcame this problem by unexpected substitution mutations around the start codon of antibodies (Nagumo et al., 2016) . These mutations destabilized the mRNA secondary structure and this somehow led to a better formation of conjugates and higher protein expression.

### 2.2. In vivo display technologies

#### 2.2.1. Bacterial surface display

The bacterial surface display technique was developed as a potential alternative to phage display (Sergeeva et al., 2006) . The use of bacteria as a display system was first reported by George Georgiou’s group in 1993 (Georgiou et al., 1993) . They first used the Lpp-OmpA’ chimera to display two specific scFvs on the outer membrane of the gram-negative bacterium E. coli. Several years later, a new approach was developed by the same group called APEx (anchored periplasmic expression) (Jeong et al., 2007) . With this second system, scFvs were displayed in the periplasmic space anchored to the inner membrane of E. coli. For isolation of antigen-specific clones, flow cytometry was used for both applications. Due to the technological shortcoming of the FACS (uflorescence activated cell sorting) of that time, library size was limited and thereby this technique was basically used for the evolution of the preexisting antibodies (Harel Inbar and Benhar, 2012) . This technique is more commonly used to display functional enzymes, antigens, and especially polypeptide libraries (up to 1011 library size) (Sergeeva et al., 2006; Liu et al., 2017) . For example, to identify peptide ligands specific for VEGF, bacteria-displayed peptide libraries were constructed and screened (Liu et al., 2017) .

#### 2.2.2. Yeast surface display

Yeast surface display was first demonstrated by Dane Wittrup’s group using Saccharomyces cerevisiae to display antibody repertoires (Harel Inbar and Benhar, 2012) . Yeast surface display is a powerful technique that allows to obtain antibodies with desired afinity, specificity, and stability. In this technique, scFvs that consist of VH and VL regions and a polypeptide linker binding them together are displayed. On yeast, scFvs are fused to the adhesion subunit of the yeast agglutinin protein Aga2p, which is bound to Aga1p via a disulfide bond and this complex attaches the scFv to the yeast cell wall and finally the desired antibody fragment is identified by FACS (Figure 1) (Feldhaus and Siegel, 2004; Chao et al., 2006; Liu et al., 2017; Mei et al., 2017) . This technique is commonly used for antibody display and it has several advantages: (i) use of FACS to monitor equilibrium activity statistics of the sample; (ii) oefring easy secretion and purification; and (iii) using yeast cells, which can perform posttranslational modification. On the other hand, it allows to display up to 109 copies of scFv, which is a limitation as compared with the other display platforms such as phage display (Chao et al., 2006; Harel Inbar and Benhar, 2012) . Also, the best known disadvantage of yeast surface display is slower growth rate and lower transformation efficiency compared to both phage and bacteria surface display techniques (Mei et al., 2017) .

#### 2.2.3. Mammalian surface display

Mammalian surface display was developed by Ira Pastan’s group in 2006 (Ho et al., 2006) . They used this technique to display an scFv library fused to the N-terminal transmembrane domain of human platelet-derived growth factor receptor (PDGFR) on the surface of HEK293T cells and were able to isolate high-afinity anti-CD22 antibodies. Mammalian cell display has powerful aspects for the isolation of scFv and whole IgG with high afinity and other specific biological functions. For instance, they can express mouse or human antibodies containing the posttranslational modifications required for some key antibody functions, and the technique can also be used to express recombinant antibody fragments that cannot be expressed in E. coli (Ho and Pastan, 2009) . However, there are only a few reports of the technique, basically due to the limitation of repertoire size (ranging between 103 and 106). Similar to other techniques, it is required to transfer genes encoding the desired proteins to proper host cells by convenient vectors and to make sure that the desired protein undergoes correct transcription and translation processes. However, due to slower proliferation rates of mammalian cells in contrast to microbial ones, it is challenging to choose cells suitable for construction of a convenient and rapid mammalian cell surface display system. HEK-293, COS, and CHO cells are the most widely used cell lines in mammalian surface display approaches. HEK-293 has particularly been preferred more than others because of its ease of transport, high yield, and native human glycosylation (Qin and Li, 2014) .

## 3. Rational design approaches

**Figure 2:**
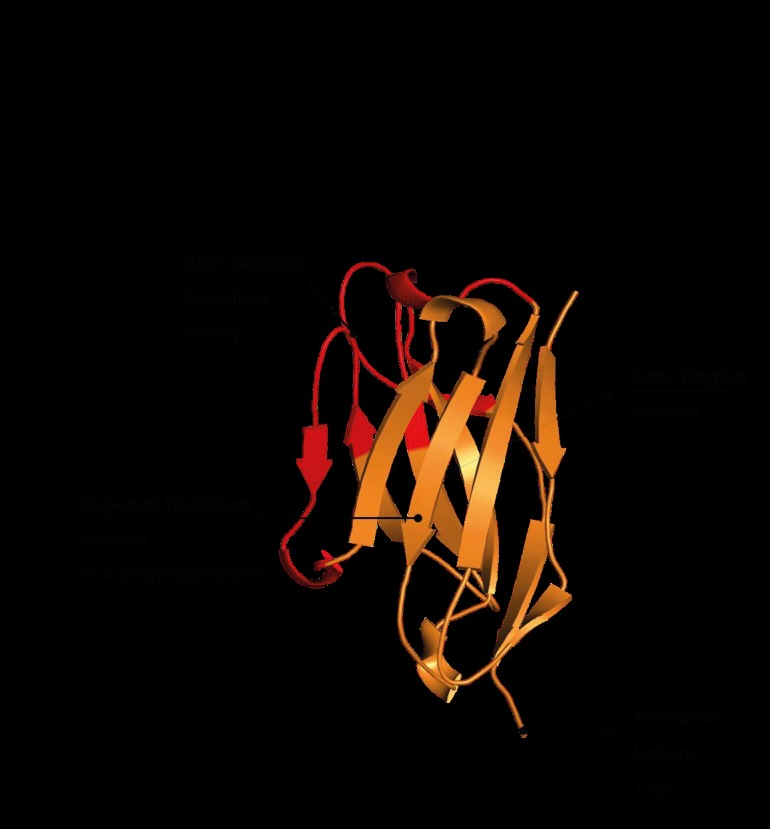
Protein engineering approaches based on antibody regions. While solubility/ aggregation can be improved by engineering exposed residues, stability can be increased by both exposed and core residues. Complementarity-determining regions (CDRs) mainly affect specificity and affinity. Tags/linkers can be added for better functionality.

Aggregation, solubility, and stability are important factors that effect the developability of an antibody. These challenges can occur during the production process due to the protein’s large complex profile and can cause reduced antigen binding afinity, immunogenic responses, and waste of resources. Aggregation/solubility and stability properties of an antibody depend on both its sequence and structure (Figure 2). It is advantageous to control these properties with rational design before in vitro and in vivo studies. Rational design methods aim to demonstrate problematic regions of protein sequences or structures. Thus, combining rational design methods and in vitro/in vivo studies enhances the chance of antibodies with better solubility and stability in the early production phase.

Intrinsic and extrinsic properties play important roles in rational design predictions (Dubay et al., 2004; Pawar et al., 2005) . Physicochemical properties of amino acids effect the antibody profile as intrinsic factors. For example, the aggregation rate of the polypeptide can be increased when the number of hydrophobic residues increases. Also, extrinsic factors such as pH, ionic strength, and temperature should be considered during sequence-based prediction (Dubay et al., 2004) . Both intrinsic and extrinsic factors might change the properties and only aggregation/ solubility can be predicted based on the sequence because the aggregation rate usually depends on amino acid properties. On the other hand, stability and afinity depend on both amino acid and structure properties.

Recent developments in rational design tools that predict problematic regions of proteins help researchers to improve antibody developability. Here, we introduce rational design web tools that can be used to improve aggregation/solubility, stability, and afinity properties based on mutagenesis.

### 3.1. Aggregation/solubility

Protein aggregation is a common problem in therapeutic antibodies and it can occur during production or storage. On the molecular level, aggregation occurs due to specific regions of a protein sequence named aggregation prone regions (APRs) that determine its aggregation rate. These APRs indicate specific charge, hydrophobicity, and secondary structural properties and lead to aggregation (Fink, 1998; Tartaglia and Vendruscolo, 2008; Agrawal et al., 2011; Elgundi et al., 2017) . Prediction of potential APRs is the key function of aggregation/solubility prediction tools.

Early studies showed that protein aggregation and stability kinetics are computable and protein sequences can be designed based on desired properties (Kamtekar et al., 1993; West et al., 1999; Worn and Pluckthun, 1999; Worn and Pluckthun, 2001) . Several prediction tools have been developed to determine the aggregation propensity of a protein (Table 1). While most of them analyze the amyloid formation, some analyze only aggregation propensity/ APRs. However, most of the tools can be used to analyze antibody fragments due to their small size. The most commonly used tools are Tango (Fernandez-Escamilla et al., 2004) , Waltz (Beerten et al., 2015) , AggreScan (Conchillo-Sole et al., 2007) , Pasta 2.0 (Walsh et al., 2014) , and Camsol Instrinsic (Sormanni et al., 2015) , which determine the aggregation propensity of an antibody based on its sequence.

**Table 1:** Protein sequence and structure-based web tools for rational design approaches.

Sequence-based Prediction Web Tools			
	Tool name	Definition	References
Aggregation/Solubility	Pasta 2.0	Predicts aggregation-prone, disordered regions	(Walsh et al., 2014)
	Tango	Evaluates the aggregation scores for each residue based on physicochemical principles	(Fernandez-Escamilla et al., 2004)
	Waltz	Computes the position-specific scores to determine aggregation-prone regions	(Beerten et al., 2015)
	Camsol Intrinsic	Gives output scores for each residue based on solubility profile of sequence	(Sormanni et al., 2015)
	AggreScan	Predicts aggregation-prone regions and estimates the effect of mutation on aggregation profile	(Conchillo-Sole et al., 2007)
	Fısh Amyloid	Identifies amyloidogenic regions in protein sequences	(Gasior and Kotulska, 2014)
	Soda	Focuses on effect of the mutations on intrinsic solubility profile of protein sequences	(Paladin et al., 2017)
	Pon-Sol	Determines the effect of amino acid variation on solubility profile	(Yang et al., 2016)
	Protein-Sol	Gives graphical outputs of highlighted lysine arginine contents and solubility profile	(Hebditch et al., 2017)
Structure Based Prediction Web-Tools			
Aggregation/solubility	Camsol StructurallyCorrected	Gives structurally corrected solubility profile to visualize poorly soluble regions on the surface, determines the proper residues for mutation	(Sormanni et al., 2015)
	AggreScan3D	Identifies the poorly soluble residues based on both position of amino acid and amino acid structure	(Zambrano et al., 2015)
Stability	ProMaya	Calculates protein stability based on differences between protein’s wild type and mutated type free energies	(Wainreb et al., 2011)
	SDM	Evaluates the stability differences between the wild type and mutated type protein structure	(Pandurangan et al., 2017)
	I-Mutant	Determines the structure and sequence-based stability changes depending on single point mutation of protein	(Capriotti et al., 2005)
	Cupsat	Uses amino acid–atom potential and torsion angle distribution information to identify changes in protein stability-based on mutations	(Parthiban et al., 2006)
Affinity	mCSM-AB	Predicts antigen–antibody affinity changes upon mutations	(Pires and Ascher, 2016)

Tango (http://tango.switchlab.org/) is the earliest aggregation prediction tool and predicts the β-sheet aggregation of a given protein sequence. It evaluates probability scores for each amino acid’s beta turn, beta sheet, alpha helix, and beta and alpha aggregation considering given extrinsic conditions (pH, temperature, ionic strength, concentration). The algorithm assumes that specific regions of protein have high aggregation propensity if they involve at least vfie consecutive residues with a probability to populate the β-aggregate state higher than 5% per residue. It was shown that Tango has a success rate of 87% , correctly predicting 155 out of 179 peptides, with 21 false positives and 3 false negatives (FernandezEscamilla et al., 2004).

Waltz (http://waltz.switchlab.org/) and Pasta 2.0 (http:// protein.bio.unipd.it/pasta2/) give highly aggregationprone/amyloid-forming regions as output. While Waltz uses a position-specific scoring matrix (PSSM) with physicochemical information to identify amyloid forming regions (Beerten et al., 2015) , Pasta 2.0 identifies amyloid forming regions by calculating the pairing energies for each pair of residues facing one another on parallel or antiparallel neighboring strands within a β-sheet (Walsh et al., 2014) .

AggreScan and Camsol are listed in two subsections of the Table because they can analyze protein aggregation propensity based on both sequence and structure information. Aggrescan (http://bioinf.uab.es/aggrescan/) calculates aggregation propensity scores for each residue in the sequence by averaging the aggregation propensity score per residue over a given length (Conchillo-Sole et al., 2007) . Aggrescan3D (A3D) (http://biocomp.chem. uw.edu.pl/A3D/) is an improved version of Aggrescan that overcomes the limitations of sequence-based analyses. A3D identifies aggregation prone residues, which are related to folded states. Also, designed/desired mutation eefcts on aggregation propensity of any protein can be determined by using A3D (Zambrano et al., 2015) .

The Camsol method can be used in two different modes, ‘Camsol Intrinsic’ and ‘Camsol Structurally Corrected’ (http://www-vendruscolo.ch.cam.ac.uk/ camsolmethod.html), to evaluate aggregation scores of any protein. Camsol Intrinsic calculates the solubility profile scores per amino acid by using the given protein sequence and identifies the regions that are poorly soluble when the score is smaller than –1. It evaluates the aggregation propensity per residue using the sequence, charge, hydrophobicity, and secondary structure propensity as intrinsic factors. Camsol Structurally Corrected analyzes the protein structure like Camsol Intrinsic but it shows the poorly soluble regions on the surface that can be used to identify suitable mutations to increase the solubility of the protein. These poorly soluble regions can also be visualized by using output structure (Sormanni et al., 2015, 2017) .

These methods can be used separately or combined to predict aggregation/solubility profiles and the combination of different methods can provide higher accuracy for mutagenesis studies. Van Der Kant et al. used only Tango for prediction of APRs as a part of a study analyzing the relationship between intrinsic aggregation propensity and the local thermodynamic stability of over 2000 antibody structures from the abYsis database (Van Der Kant et al., 2017) . Wang et al. combined Tango with structure-based methods to predict APRs in antibody sequences based on 29 published Fab-antigen complexes (Wang et al., 2010) . They tested two different thresholds and they found that Tango was more than 92% correct in their experimental validation studies. In another study, estimations of Tango, Aggrescan, and Pasta 2.0 were used to identify APRs that were mostly confirmed by experimental results (Yageta et al., 2015) .

Lately several sequence-based aggregation propensity prediction tools have also been developed. Gasior and Kotulska proposed a classification method called Fish Amyloid (http://comprec-lin.iiar.pwr.edu.pl/) that is able to recognize amyloidogenic fragments based on welldefined patterns of residue distribution and cooccurrence of position-specific amino acids in protein sequences (Gasior and Kotulska, 2014) . Fish Amyloid was trained on different lengths of sequences and oefred good potential for prediction. PonSol (http://structure.bmc.lu.se/PON-Sol) determines the eefct of amino acid variations solubility profiles. The tool uses 443 amino acid substitutions from 71 proteins and these amino acid substitutions are classified as increasing, decreasing, and not aefcting solubility (Yang et al., 2016) . Protein-Sol (https://protein-sol.manchester. ac.uk/) is another recent sequence-based prediction tool that uses datasets of Escherichia coli protein solubility for comparison and calculates 35 sequence-based properties. The tool gives graphical output of predicted solubility, fold propensity, and net segment charge. Predicted solubility scales from 0 to 1 and more than 0.45 solubility scores are accepted as soluble. Also, lysine and arginine contents are highlighted for modifying protein solubility (Hebditch et al., 2017) . Soda (http://protein.bio.unipd.it/soda/) predicts the protein solubility changes based on calculations of several physicochemical properties for given mutations. The method compares the mutant type and wild type profile properties and estimates the changes. Soda provides convenience for different types of variations such as point mutation, deletion, or insertion (Paladin et al., 2017) .

**Figure 3:**
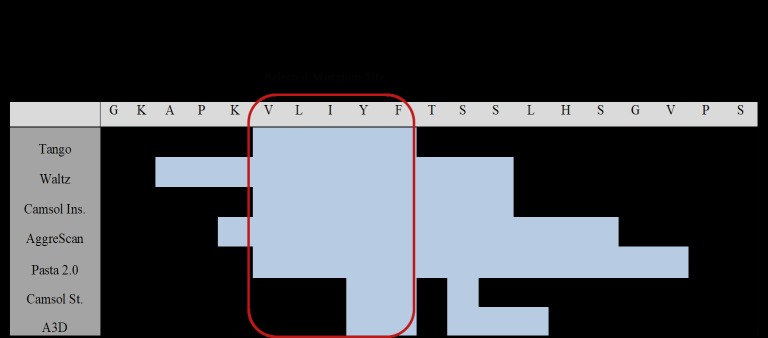
A case study for web tools. Several tools introduced in this review were used to determine aggregation-prone regions of an scFv sequence used in our lab. Blue highlighted regions are outputs of tools as aggregation prone regions. Each web tool has a different threshold, which was not shown in this figure. Mutation site is selected according to common predicted regions of different tools (at least 6 of 8 tools gave same residues as aggregation-prone).

As a case study, an scFv sequence used in our lab was analyzed with some of the sequence-based tools introduced above (Figure 3). The full scFv sequence was given as input. As output, every residue had an aggregation/solubility score based on the tool’s calculation and they were highlighted as aggregation-prone according to the tool’s corresponding thresholds. We determined multiple regions of the scFv as aggregation-prone (at least 6 of 8 tools gave predicted aggregation-prone residues). One of those regions is shown as an example in Figure 3. Our future mutations will be focused on those regions to improve the biophysical characteristics of our protein.

### 3.2. Stability

Protein stability can be predicted by calculating the change in the Gibbs free energy due to substitution of an amino acid and more negative values of free energy present better stability (Thiltgen and Goldstein, 2012) . Different approaches can be used for prediction of protein stability, such as physical, statistical, empirical, and/or machine learning methods. While the first three approaches are limited and are more time- and cost-intensive, machine learning methods can quickly perform predictions based on input mutation, protein sequence, and structural information at the same time (Capriotti et al., 2004; Cheng et al., 2006) .

Several web-based tools were developed to predict protein stability. ProMaya (http://bental.tau.ac.il/ ProMaya/) calculates the stability free energy change upon mutations by combining a collaborative filteringbased algorithm (CF) and random forest regression. The tool uses different available datasets of mutations in the same and different positions. ProMaya suggests that using known free energy values of mutations at a specific position corrects the prediction of free energy differences for other mutations (Wainreb et al., 2011) .

SDM (http://marid.bioc.cam.ac.uk/sdm2) evaluates the stability change between the wild type and mutant protein by using a conformationally constrained environment-specific substitution table (ESST). The method analyzes the amino acid alteration with specific structural parameters based on residue packing density and the ESST. The webserver gives predicted stability difference scores interpreted as reduced, induced, or unaffected stability (Pandurangan et al., 2017) .

I-Mutant (http://gpcr2.biocomp.unibo.it/cgi/ predictors/I-Mutant3.0/I-Mutant3.0.cgi) predicts protein stability changes based on a support vector machine and allows users to use protein structure or sequences for prediction. It was shown that I-Mutant has an accuracy of 77%–80% for the dataset derived from ProeThrm (Bava et al., 2004; Capriotti et al., 2005) .

Cupsat (http://cupsat.tu-bs.de/) uses atom potential and torsion angle distribution information of amino acids to identify protein stability free energy change upon mutations. The tool analyzes the protein structure and gives information about mutation site, solvent accessibility, and torsion angle and whether the mutated amino acid has suitable torsion angles or not. It was shown that Cupsat achieved 80% prediction success for both thermal and chemical stability (Parthiban et al., 2006) .

### 3.3. Afinity/specificity

If afinity improvement is desired, in vitro/vivo methods explained Section 2 of this review can be used. eThre are many available afinity maturation strategies based on directed evolution methods. Generally, mutations in complementarity-determining regions (CDRs) for improving antigen–antibody afinity cannot be predicted by using rational design approaches because it is hard to estimate the dynamic antigen–antibody complex structure. However, there is a newly developed tool called mCSM-AB (http://biosig.unimelb.edu.au/mcsm_ab/) that uses free energy change upon mutation and estimates the afinity change. In the tool, a negative sign means that the selected mutation reduces afinity and a positive sign means that the selected mutation increases afinity. It is important to know that this tool allows users to select more than one mutation (Pires and Ascher, 2016) .

## 4. Discussion

The main aims of protein engineering approaches are usually to improve afinity/specificity or to prevent aggregation and increase solubility and stability while not changing afinity/specificity. Although there are some trade-ofs during these processes, there are many successful examples in the literature that improved the biophysical characteristics of antibodies.

Enever et al. used a new approach called phage display stress selection to screen for more stable human nanobodies (Enever et al., 2015) . Their goals were to improve thermodynamic stability and to make nanobodies resistant to aggregation. They generated error-prone PCR phage libraries and subjected these libraries to various stress conditions. Stress conditions were related to temperature (incubation at 50–80 °C for various amounts of time), pH (incubation at pH 3.2 for various amounts of time), and protease (incubation with trypsin, elastase, leucozyme). Selection results revealed that beneficial mutations (both on CDRs and framework residues) were common to most of the stress conditions. This means that antibodies tend to mutate generic amino acids to improve their biophysical properties.

Dudgeon et al. introduced a general strategy to improve biophysical properties of antibody variable domains (Dudgeon et al., 2012) . They identified specific positions in CDR regions (28, 30–33, 35 in VH and 24, 49–53, 56 in VL) and mutated those to aspartate or glutamate. This strategy led to increased aggregation resistance, which is advantageous for both diagnostic and therapeutic applications. Although most of those mutations were located in CDR regions, they showed that binding performances were not significantly affected for nearly half of the mutants.

Courtois et al. rationally designed a biobetter drug candidate by mutating or engineering aggregation-prone residues of a Fab fragment (Courtois et al., 2016) . They removed aggregation-prone residues by single point mutations (hydrophobic residues to charged aspartate or lysine) and found that stability increased up to 4-fold. They also added a glycosylation site near aggregationprone regions to increase solubility and up to 3-fold increases in stability were obtained. Most importantly, these engineering approaches did not alter binding to the target.

Before designing mutations to decrease aggregation and/or increase stability of antibodies, three important points should be considered carefully: (i) CDR regions of the sequence should not be selected for mutation although they have high predicted scores because they are usually important for antigen binding and afinity/specificity might be impaired. (ii) Exposed hydrophobic amino acids are widely known to contribute to aggregation, and those residues should be considered first for mutation. They are preferentially mutated to hydrophilic, even charged amino acids such as aspartate, glutamate (Dudgeon et al., 2012) , or lysine (Courtois et al., 2016) to circumvent aggregation problems. (iii) Designed mutations should also be compared with a natural repertoire because mutating a residue to its naturally conserved amino acid might improve its properties. The abYsis database is a web-based tool that integrates sequence data from the European Molecular Biology Laboratory European Nucleotide Archive (EMBL-ENA) and structure data from the Protein Data Bank (PDB). The abYsis database can be used to determine location-specific amino acid distribution of the natural repertoires of different organisms (Swindells et al., 2017) .

It is important to note that there could be some tradeofs while improving the desired properties of an antibody (solubility, stability, afinity). Thus, the designed change should be considered for all properties. For example, while a mutation increases the solubility, it might also decrease stability at the same time. Afinity maturation can lead to a better binder but this higher afinity antibody might fail in the development phase due to its poor biophysical characteristics. It is important to keep in mind that tradeofs can occur while improving antibody fragments and one should design their computational/experimental setup accordingly.

## Acknowledgments

We would like to thank the İzmir Biomedicine and Genome Center and YÖK (Council of Higher Education) 100/2000 fellowship program for funding our research group. We thank Hasan Buğra Çoban for his valuable input during writing process. We thank all of our research group members for carefully reviewing this article before submission.

Bain B, Brazil M (2003). Adalimumab. Nature Reviews Drug Discovery 2: 693-694.
